# Hydrochar and pyrochar enhanced soil fungal community diversity in a *Quercus acutissima* plantation under severe nitric acid-type acid rain stress

**DOI:** 10.3389/fpls.2026.1821765

**Published:** 2026-05-28

**Authors:** Yan Wang, Shushu Yao, Haibo Hu, Xiaopeng Xu, Xiaoxiao Liu, Peng Cui, Danyan Chen, Yuanhao Liu, Yuanyuan Feng

**Affiliations:** 1 Co-Innovation Center for Sustainable Forestry in Southern China, College of Forestry and Grassland, Nanjing Forestry University, Nanjing, China; 2 School of Agriculture, Yunnan University, Kunming, China; 3 Department of Applied Physics and Electronics, Umea University, Umea, Sweden; 4 College of Horticulture, Jinling Institute of Technology, Nanjing, China

**Keywords:** hydrochar, nitric acid-type acid rain, plantation, pyrochar, soil fungal community

## Abstract

Intensifying nitric acid-type acid rain (NAR) poses a serious threat to terrestrial ecosystem functioning. Biochar (BC), as a soil amendment, has the potential to mitigate soil degradation caused by acid deposition. However, under severe NAR stress, the mechanisms by which BC affects soil fungal communities in plantations remain largely unexplored. In this study, a field experiment simulating severe NAR was conducted in a *Quercus acutissima* plantation. Two BC types, including pyrochar (PC) and hydrochar (HC), were applied to the soil to examine their effects on soil fungal community composition and diversity. The results showed that the PC application significantly increased the relative abundance of Basidiomycota in soil by 20.92%, whereas HC significantly decreased it by 57.83% under severe NAR stress. Linear discriminant analysis Effect Size results suggest that PC and HC addition promoted the enrichment of specific fungal taxa, such as *Amanitaceae*, *Penicillium*, and *Strophariaceae*. Additionally, PC and HC increased the soil fungal Shannon index by 19.81% and 42.45%, respectively, under severe NAR stress. Furthermore, redundancy analysis revealed that changes in soil fungal community structure induced by BC application under severe NAR were mainly driven by soil microbial biomass. Our findings demonstrate that HC is more effective than PC in alleviating severe NAR stress on soil fungal communities in plantations, providing a scientific basis for the resource utilization of BC and the ecological restoration of plantations in NAR-affected regions.

## 1 Introduction

Acid rain (AR) is one of the most serious ecological and environmental challenges worldwide (Grennfelt et al., 2020). China, Europe and North America are the three major regions with concentrated AR occurrence, and the area affected by AR in China has exceeded 40% of the national territory (Kulshrestha, 2020; Lv et al., 2014). With ongoing urbanization and industrialization, emissions of sulfur dioxide (SO_2_) have been partially controlled, whereas emissions of nitrogen oxides and ammonia have continued to increase (Duan et al., 2016; Zhou et al., 2022). This shift in the emission structure has led to a continuous decline in the molar ratio of sulfate (SO_4_^2-^) to nitrate (NO_3_^-^) in precipitation (Fang et al., 2011). Over the past four decades, the SO_4_^2-^/NO_3_^-^ ratio in precipitation in the Yangtze River Delta region of China has decreased from 7.5 to around 2.0, and the dominant AR type has gradually shifted from sulfate-type to nitric acid-type, further evolving towards severe nitric acid-type AR (NAR) (Li et al., 2021a). Severe NAR exerts particularly strong disturbance effects on terrestrial ecosystems (Li et al., 2021b). Compared with mild AR, severe NAR induces more pronounced soil acidification and accelerates soil degradation (Chen et al., 2013). Therefore, developing effective strategies for remediating soil acidification is of great and pressing importance for maintaining regional ecological security and promoting the sustainable development of terrestrial ecosystems.

Biochar (BC), as a low-cost and efficient soil amendment, has shown considerable potential for mitigating soil degradation caused by AR stress. The mainstream BC production technologies currently include high-temperature pyrolysis and hydrothermal carbonization, whose products are referred to as pyrochar (PC) and hydrochar (HC), respectively (Kambo and Dutta, 2015). PC is produced by thermally decomposing dry biomass under oxygen-limited conditions at 300-650 °C, characterized by high porosity, cation exchange capacity and proportion of aromatic carbon (C) (Heikkinen et al., 2019). These properties enable PC to optimize the soil C: N ratio and create microhabitats suitable for microorganisms (Schnee et al., 2016), thereby enhancing the diversity of soil microbial communities. In contrast, HC is produced from wet biomass via hydrothermal carbonization at 180-260 °C under autogenous pressure (Kambo and Dutta, 2015). The surface of HC is rich in oxygen-containing functional groups and exhibits higher chemical reactivity, providing readily available energy substrates for soil microorganisms, which can enhance microbial metabolic activity and increase soil microbial biomass (Palansooriya et al., 2019).

As an essential component of soil microorganisms, fungal community structure and metabolic processes are highly sensitive to changes in the soil environment. A previous study has reported that soils tend to exhibit high N:P ratios and pronounced acidification, leading to an increase in the relative abundance of acid-tolerant Ascomycota and a decrease in the relative abundance of Basidiomycota under NAR stress (Zhou et al., 2023). Liu et al. (2017) found that NAR suppressed litter decomposition, reduced the availability of C substrates for fungi, and significantly decreased total soil phospholipid fatty acids. Several studies have demonstrated that BC could increase soil pH and nutrient availability, thereby enhancing soil fungal diversity (Li et al., 2020). In particular, the well-developed pore structure of PC provided the colonization microenvironment for mycorrhizal fungi (e.g., ectomycorrhiza and arbuscular mycorrhiza), effectively maintaining soil fungal community diversity (Heikkinen et al., 2019; Li et al., 2023b). By comparison, the surface of HC retains more oxygen-containing functional groups and labile C, which may confer unique advantages in promoting microbial energy metabolism and nutrient turnover (Kravchenko et al., 2024). However, the differential effects of PC and HC on soil fungal community structure under severe NAR stress remain largely unexplored. We hypothesized that the application of PC and HC could alleviate the negative impacts of severe NAR on soil fungal community structure, and HC might be more effective than PC in maintaining fungal community diversity.

Herein, a field experiment simulating severe NAR was conducted in a *Quercus acutissima* plantation. Two BC types (i.e., PC and HC) were applied to the soil to examine their effects on soil fungal community composition and diversity. The main objectives were to: (1) examine the effects of BC on soil fungal community composition and diversity in a *Q. acutissima* plantation under severe NAR stress; (2) compare the responses of soil fungal communities to PC and HC application under severe NAR; and (3) elucidate the mechanisms by which PC and HC drive the restructuring of soil fungal communities under severe NAR stress. The findings of this study will provide a scientific reference for ecological restoration and soil quality improvement in plantations affected by NAR.

## 2 Materials and methods

### 2.1 Study area overview

The study was conducted at the Yangtze River Delta Forest Ecosystem Research Station (32°7′49″N, 119°12′7″E) in Zhenjiang, Jiangsu Province, China. The region has a typical subtropical monsoon climate. The mean annual air temperature is 15.1 °C, and the mean annual precipitation is 1184.3 mm. The mean precipitation pH is 5.15 (Li et al., 2021a). The study area is located in hilly terrain at an elevation of approximately 180 m. The soil has the following properties: bulk density, 1.4 g cm^-3^; soil water content, 14.6%; pH, 5.0; soil organic carbon (SOC), 33.5 g kg^-1^; and total nitrogen (TN), 3.2 g kg^-1^. The overstory vegetation consists mainly of a *Q. acutissima* plantation. The shrub layer is sparsely distributed and is dominated by *Rosa multiflora* Thunb., *Fortunearia sinensis* Rehd. et Wils. and *Ilex cornuta* Sims. The dominant species in the herb layer include *Parthenocissus tricuspidata* (Siebold & Zucc.) Planch., *Ophiopogon japonicus* (L.f.) Ker Gawl. and *Trachelospermum jasminoides* (Lindl.) Lem (Feng et al., 2026).

### 2.2 Preparation of PC and HC

The PC used in this experiment was produced from naturally senesced *Q. acutissima* leaves collected in the Xiashu Forest Farm, Zhenjiang, China. Briefly, the leaf litter was air-dried, then placed in a muffle furnace and pyrolyzed under oxygen-limited conditions at 500 °C for 2 h. After the furnace cooled to room temperature, the solid product was ground and passed through a 2 mm sieve. The resulting black powdery PC was sealed and stored.

The HC was prepared following the method described by Xu et al. (2025). Finely ground *Q. acutissima* leaf litter (<2 mm) was mixed with deionized water at a solid-to-liquid ratio of 1:10 (w/v) and transferred into a high-pressure hydrothermal reactor. The reactor was sealed and heated to 260 °C and maintained at this temperature under autogenous pressure for 1 h. After the reaction, the reactor was allowed to cool naturally to room temperature. The solid product was then oven-dried, sieved through a 2 mm mesh, and stored in amber glass bottles in the dark. The Scanning Electron Microscope micrographs and basic properties of PC and HC were detailed in **Supplementary Figure 1** and **Supplementary Table 1**.

### 2.3 Experimental design

The experiment was established in a pure *Q. acutissima* plantation in the Xiashu Forest Farm, with a stand age of approximately 70 years as determined from the management records, stand density of 430 trees ha^-1^, and mean diameter at breast height of 28.6 cm. A randomized complete block design was adopted. Twelve plots (3 m × 3 m) were randomly established within the stand, each separated by a 3 m buffer zone. To prevent lateral flow of NAR solution, polyvinyl chloride boards (30 cm high, 0.5 cm thick) were vertically installed around each plot, with 25 cm buried in the soil and 5 cm above the soil surface. Four treatments were set up, each with three replicates: (1) control (CK): no BC addition, sprayed with deionized water (pH ≈ 6.5); (2) AR: sprayed only with simulated severe NAR; (3) PC combined with NAR (PC-AR): sprayed with simulated severe NAR and amended with PC; and (4) HC combined with NAR (HC-AR): sprayed with simulated severe NAR and amended with HC.

Following the method of Lv et al. (2014) and Liu et al. (2018), stock solutions of 0.5 mol L^-1^ H_2_SO_4_ and 0.5 mol L^-1^ HNO_3_ were mixed at a molar ratio of 1:5 (SO_4_^2-^:NO_3_^-^), and then diluted with deionized water to pH 2.5 to obtain the severe NAR solution (Zhou et al., 2023). The application rate of simulated NAR was determined according to local rainfall records from 2011–2021 at the ecological station. From June 2021 to June 2022, the simulated NAR (or deionized water in CK) was sprayed once per month in the middle of each month, with the volume equal to the corresponding monthly mean precipitation (Liu et al., 2022). In June 2021, PC and HC were evenly applied to the soil surface in the PC-AR and HC-AR plots, respectively, at a rate of 0.5 kg m^-2^. No tillage or other soil disturbance was carried out during the experimental period.

### 2.4 Soil chemical property determination

Soil samples were collected on 30 June 2022. In each plot, five subsamples of topsoil (0–10 cm) were collected using a five-point composite sampling method and thoroughly mixed (Wang et al., 2020). Roots and gravel were removed, and the samples were passed through a 2 mm sieve. Each composite sample was then divided into two portions. One was air-dried for the determination of soil physicochemical properties. The other one was stored at -80 °C for analysis of soil enzyme activity, microbial biomass and fungal community.

SOC and TN were determined using a elemental analyzer (Vario EL III, Elementar, Germany). Total phosphorus (TP) was estimated using inductively coupled plasma optical emission spectroscopy (ICP-OES, PerkinElmer Optima 8000, USA). Soil microbial biomass C (MBC), N (MBN), and P (MBP) were determined by the chloroform fumigation-extraction method (Li et al., 2023a). Soil enzyme activities, including β-1,4-glucosidase (BG), N-acetyl-β-D-glucosaminidase (NAG), leucine aminopeptidase (LAP), and acid phosphatase (AP), were measured using microplate fluorometric assays (German et al., 2011).

### 2.5 Microbial high-throughput sequencing

Total DNA was extracted from soil samples using the E.Z.N.A.^®^ Soil DNA Kit (Omega Bio-tek, Norcross, USA) according to the manufacturer’s instructions. DNA integrity was checked by 1% agarose gel electrophoresis, and samples with clear main bands and no obvious degradation (RIN > 7.0) were retained. The fungal internal transcribed spacer 1 (ITS1) region was amplified using the fungal-specific primers ITS1F (5′−CTTGGTCATTTAGAGGAAGTAA−3′) and ITS2R (5′−GCTGCGTTCTTCATCGATGC−3′). A unique 6 bp barcode was attached to each sample for sequence identification. PCR products were verified by 2% agarose gel electrophoresis, and the target band (~300 bp) was excised and purified using the AxyPrep DNA Gel Extraction Kit (Axygen Biosciences, USA). The purified DNA was eluted in 30 μL nuclease-free water. After quantification with a Qubit^®^ 3.0 fluorometer (Life Invitrogen), sequencing libraries were constructed, and paired-end sequencing (2 × 250 bp) was performed on an Illumina NovaSeq 6000 platform (Nanjing GenePioneer Co., Ltd., China).

For fungal community analysis, raw paired-end reads were first merged using PANDAseq (v2.11) with a minimum overlap length of 20 bp and a maximum mismatch rate of 10%. The merged sequences were then quality-filtered with PRINSEQ (v0.20.4) to remove low-quality reads (mean Phred score < 20), reads containing ambiguous bases, and reads shorter than 200 bp. The filtered reads were processed with the DADA2 pipeline for quality filtering, denoising, error correction, paired-end merging, and chimera removal, yielding a set of non-redundant amplicon sequence variants (ASVs) (Callahan et al., 2016). Taxonomic assignment of ASVs was performed against the UNITE database. Alpha diversity indices and beta diversity (Bray-Curtis distance) were then calculated for subsequent community analysis. Fungal ITS sequences were identified using the UNITE database (version 10.0, https://unite.ut.ee). The sequencing data have been deposited in the China National Center for Bioinformation (CNCB) database under accession number OMIX015503 (https://ngdc.cncb.ac.cn/omix).

### 2.6 Statistical analysis

All statistical analyses were conducted using SPSS v21.0 and R v4.3.1. One-way analysis of variance (ANOVA) was used to test for differences among treatments, and Tukey’s HSD *post hoc* test was applied for *post hoc* comparisons at *P* < 0.05. Heatmaps for the top six abundant fungal phyla and the top twenty abundant genera, as well as a biclustering heatmap for the fifty most abundant genera, were generated using the “pheatmap” package in R. Relative abundance data were standardized by Z-score transformation, and the color gradient represented relative abundance levels. Venn diagrams showing shared and unique ASVs among the four treatments were drawn using the “VennDiagram” package. Linear discriminant analysis effect size (LEfSe) with an LDA threshold of 3.0 was used to identify indicator taxa that significantly differed among treatments. Fungal community beta diversity was assessed based on Bray-Curtis distance matrices calculated with the “vegan” package in R, and visualized using principal coordinate analysis (PCoA). The contributions of soil chemical properties to variation in soil fungal community composition were evaluated by canonical correspondence analysis (CCA) using Canoco 5. Pearson correlation analysis was used to examine relationships between soil chemical properties and fungal alpha diversity indices. The drivers of variation in soil fungal community diversity were further analyzed by redundancy analysis (RDA) combined with Monte Carlo permutation tests (999 permutations, *P* < 0.01).

## 3 Results

### 3.1 Effects of BC application on soil chemical properties under severe NAR

**Figure 1:**
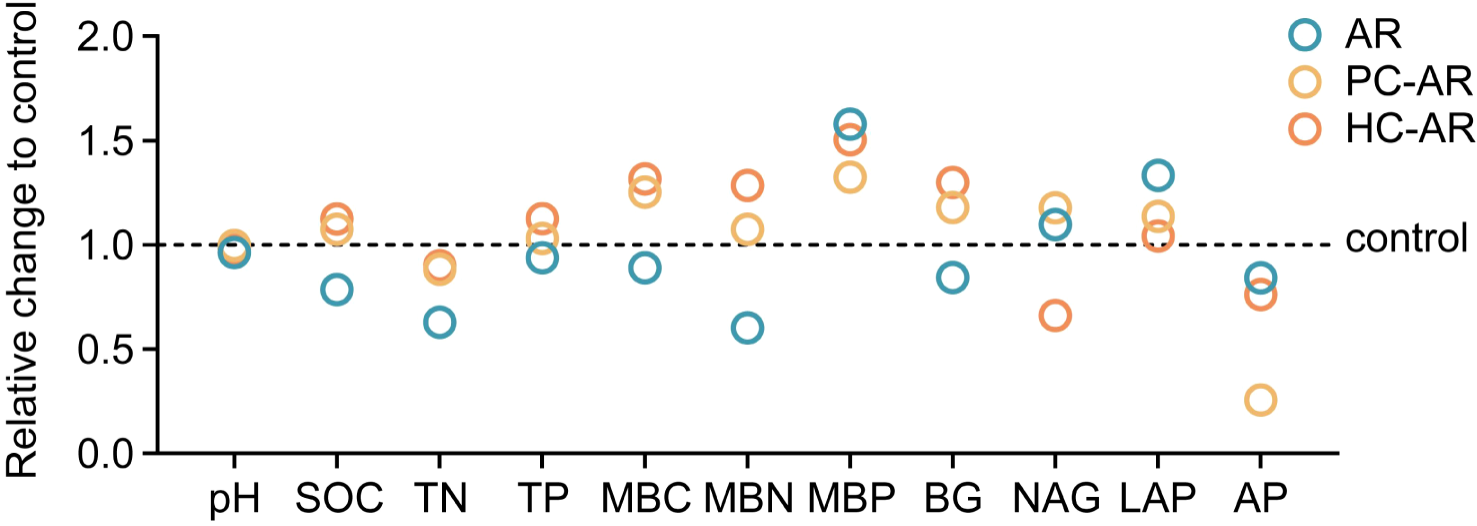
Relative changes in soil biochemical properties compared to the control under different treatments. The horizontal dashed line represents the control group values. SOC, soil organic carbon; TN,total nitrogen; TP, total phosphorus; MBC, microbial biomass carbon; MBN, microbial biomass nitrogen; MBP, microbial biomass phosphorus; BG,β-glucosidase; NAG, N-acetyl-β-D-glucosaminidase; LAP, leucine aminopeptidase; AP, acid phosphatase; AR, acid rain; PC, pyrochar; HC, hydrochar; PC-AR,PC combined with AR. HC-AR,HC combined with AR. CK, Control without PC, HC or AR.

**Figure 1** shows that the soil pH in the AR group was significantly decreased by 3.69% compared with CK, while the soil pH in the PC−AR group was significantly increased by 3.60% relative to AR (*P* < 0.05). Compared with CK, AR significantly reduced SOC content by 21.49% (*P* < 0.05). Nevertheless, SOC contents in PC-AR and HC-AR were significantly increased by 36.89% and 43.21%, respectively, relative to AR (*P* < 0.05). Soil TN content in AR was significantly decreased by 37.25% compared with CK, whereas PC-AR and HC-AR increased TN by 40.63% and 43.75% compared with AR. Soil TP content was the highest in HC-AR, higher than that in AR by 20.00% (*P* < 0.05). For microbial biomass, soil MBC and MBN content in HC-AR were significantly higher than those in AR by 40.75% and 113.55%, respectively (*P* < 0.05). The MBC content in PC-AR was also higher than that in AR by 36.84%. Soil MBP of AR and HC-AR was significantly increased by 57.89% and 50.40%, respectively, in comparison with CK (*P* < 0.05). Soil BG activity was the highest in HC-AR, 54.17% higher than that in AR (*P* < 0.05). In contrast, soil NAG activity in HC-AR was significantly lower than that in CK, AR and PC-AR by 33.87%, 39.70% and 43.84%, respectively (*P* < 0.05). Regarding soil AP activity, PC-AR showed the most pronounced response. Compared with CK, AP activity in PC-AR and HC-AR was significantly reduced by 74.43% and 23.90%, respectively (*P* < 0.05). In addition, AP activity in PC-AR was 69.65% and 66.40% lower than that in AR and HC-AR, respectively (*P* < 0.05).

### 3.2 Effects of BC application on soil fungal community composition under severe NAR

**Figure 2:**
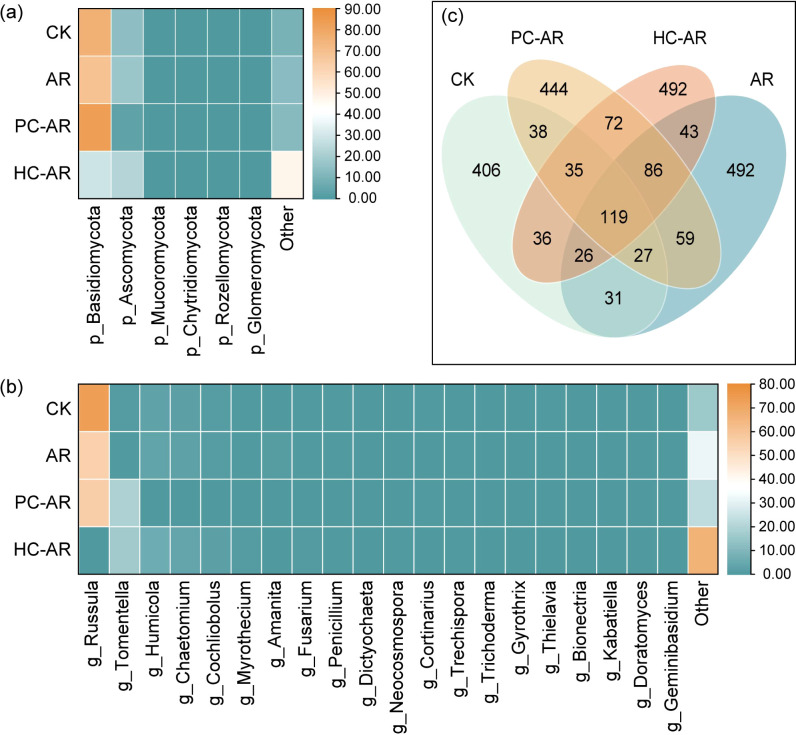
Soil fungal community compositions under different treatments. The relative abundance of fungal taxa at the **(A)** phylum and **(B)** genus levels. The color gradient represents the percentage of relative abundance. **(C)** The number of shared and unique fungal amplicon sequence variants (ASVs) among all treatments. AR, acid rain; PC, pyrochar; HC, hydrochar; PC-AR, PC combined with AR. HC-AR, HC combined with AR. CK, Control without PC, HC or AR.

According to taxonomic annotation, all fungal ASVs were assigned to 7 phyla and 272 genera. At the phylum level, Basidiomycota (29.07%–83.35%) and Ascomycota (3.67%–23.15%) were the most abundant, together accounting for 70.94% of all soil fungal sequences and representing the dominant components of the soil fungal community, followed by Mucoromycota, Chytridiomycota, Rozellomycota, and Glomeromycota (**Figure 2A**). Under severe NAR stress, the addition of PC increased the relative abundance of Basidiomycota in soil by 20.92%, whereas the addition of HC significantly decreased the relative abundance of Basidiomycota by 57.83% (*P* < 0.05). Basidiomycota, Ascomycota, and Mucoromycota were the dominant fungal phyla across all treatments. Glomeromycota was detected only in PC-AR, while Rozellomycota was exclusive to HC-AR. Additionally, Chytridiomycota was observed only in PC-AR and HC-AR, with the majority (81.25%) distributed in HC-AR. The relative abundance of dominant fungal phyla in each treatment is shown in **Supplementary Table 3**.

The top 20 fungal genera in terms of relative abundance across all treatments are shown in **Figure 2B**. At the genus level, *Russula* (0.05%–73.53%), *Tomentella* (0.59%–19.31%), *Chaetomium* (0.04%–3.87%), and *Cochliobolus* (0.07%–2.53%) were relatively abundant in all treatments, together accounting for 40.78% of the total fungal genera in soil. The dominant genera differed among treatments. In CK, *Russula* (73.53%) was the most dominant genus, followed by *Humicola* (3.11%), *Chaetomium* (2.34%), and *Cochliobolus* (1.17%). In AR, *Russula* (55.44%) was also the most dominant genus, followed by *Humicola* (3.77%), *Chaetomium* (2.84%), *Cochliobolus* (1.40%), and *Amanita* (1.14%). In terms of PC-AR, *Russula* (56.29%) remained the dominant genus, with *Tomentella* (19.31%) as the subdominant genus. In HC-AR, *Tomentella* (16.93%) became the dominant genus, followed by *Humicola* (6.17%), *Chaetomium* (3.87%), and *Cochliobolus* (2.53%). Under severe NAR stress, PC and HC significantly increased the relative abundance of *Tomentella* (*P* < 0.05), whereas HC significantly reduced the relative abundance of *Russula* (*P* < 0.05). Additionally, the relative abundance of *Penicillium* in HC-AR was significantly higher than that in PC-AR (*P* < 0.05).

As shown in **Figure 2C**, the total number of ASVs in CK, AR, PC-AR, and HC-AR was 718, 883, 880, and 909, respectively. The number of unique ASVs was 406 in CK, 492 in AR, 444 in PC-AR, and 492 in HC-AR, accounting for 56.55%, 55.72%, 50.45%, and 54.13% of total ASVs in each corresponding group, respectively. A total of 119 core ASVs were shared across the four groups. Under severe NAR stress, the number of ASVs in the HC-AR treatment was 48 higher than that in the PC-AR treatment (**Supplementary Table 2**). Statistics of unique and shared ASVs among different treatments are provided in **Supplementary Table 2**.

### 3.3 Comparison of soil fungal community differences induced by BC application under severe NAR stress

**Figure 3:**
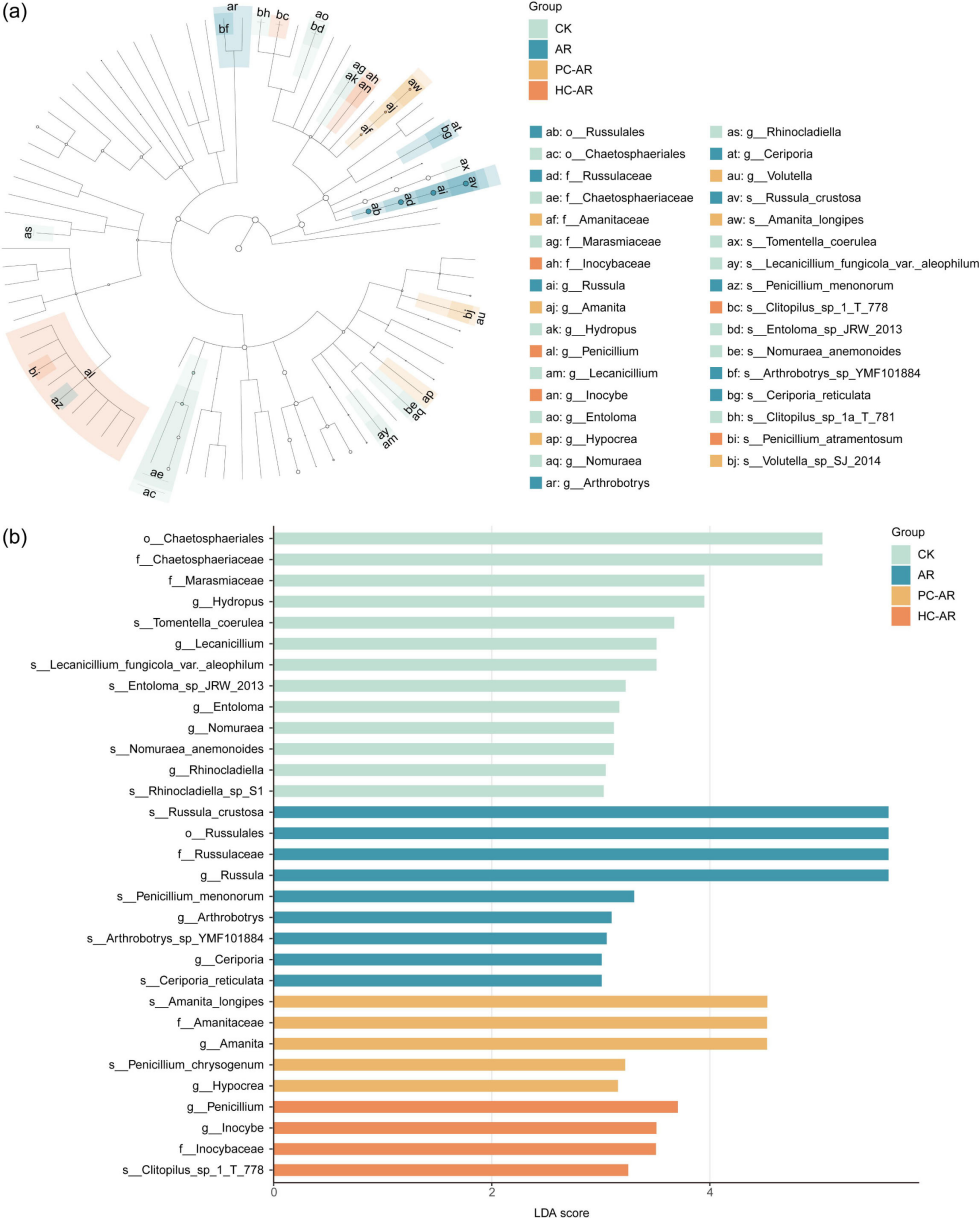
Linear discriminant analysis effect size (LEfSe) analysis identifying differentially abundant fungal taxa across treatments. **(A)** The phylogenetic distribution of fungal biomarkers. Circles represent taxa from phylum to species, with colors indicating the group where the taxon is significantly enriched. **(B)** Fungal taxa with significant differential abundance (LDA score > 3.0). AR, acid rain; PC, pyrochar; HC, hydrochar; PC-AR, PC combined with AR. HC-AR, HC combined with AR. CK, Control without PC, HC or AR.

As shown in **Figure 3A**, significant differential nodes were detected at four taxonomic levels from order to species, including 2 orders, 5 families, 13 genera, and 13 species. With an LDA threshold of > 3.0, a total of 31 fungal taxa with significant intergroup differences were identified as biomarkers. The highest number of biomarkers was enriched in CK (n = 13), followed by AR (n = 9), PC-AR (n = 5), and HC-AR (n = 4) (**Figure 3B**). In CK, the specific biomarkers were mainly concentrated in Ascomycota, including *f_Chaetosphaeriaceae* (LDA = 5.03) and *g_Lecanicillium* (LDA = 3.51). In AR, the specific biomarkers were mainly distributed in Basidiomycota, such as *Russulales* (LDA = 5.64), and in Mucoromycota, such as *g_Arthrobotrys* (LDA = 3.10). *Amanitaceae* (LDA = 4.52) and *Hypocrea* (LDA = 3.16), belonging to Basidiomycota and Ascomycota, respectively, were highly enriched and specific to PC-AR. In HC-AR, the specific biomarkers included *g_Penicillium* (Ascomycota) and *f_Inocybaceae* (Basidiomycota), with LDA values of 3.71 and 3.50, respectively.

**Figure 4:**
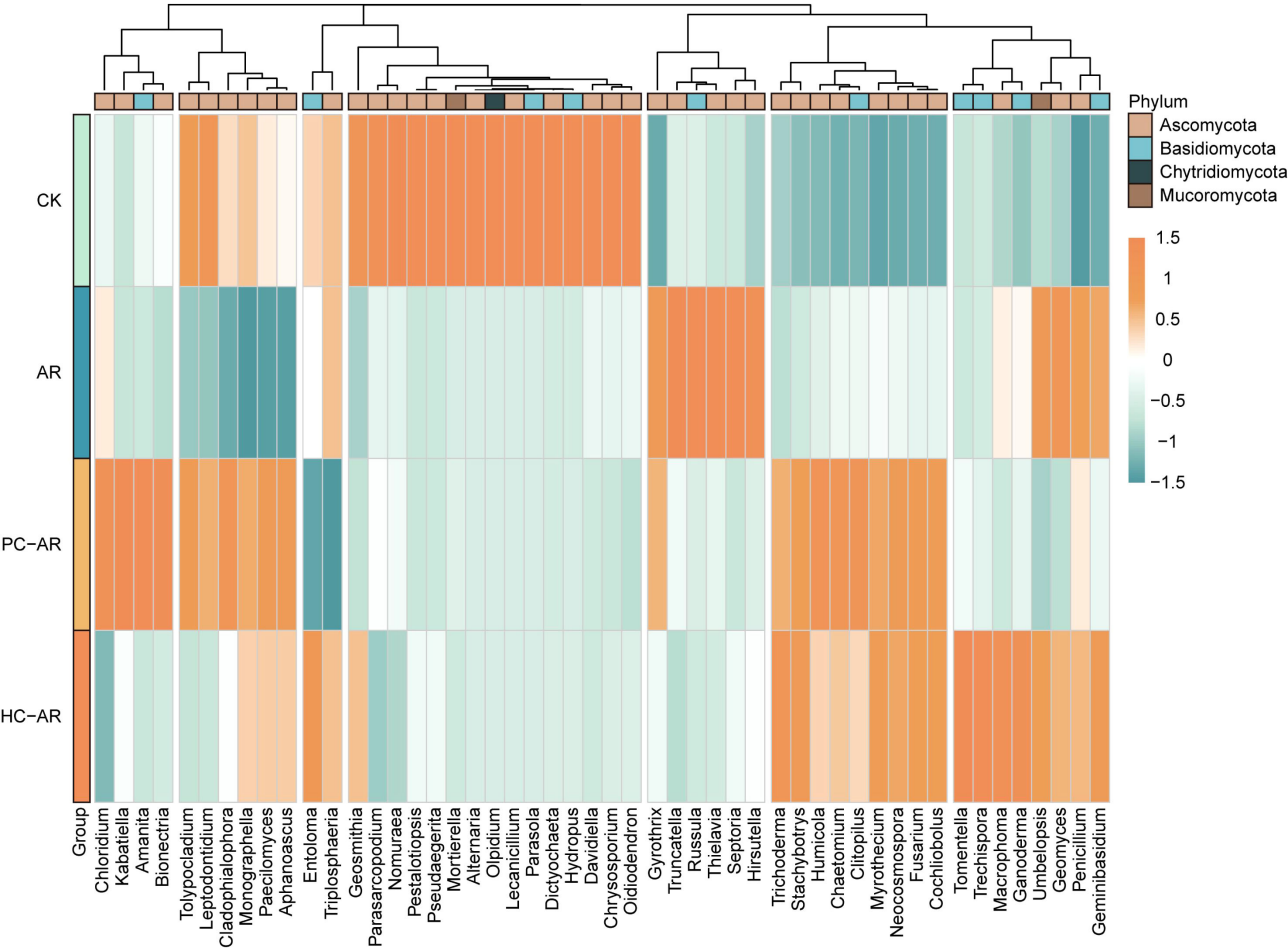
Dominant fungal genera across different treatments. The heatmap displays the relative abundance of fungal genera, standardized as Z-scores. The color gradient ranges from green (low abundance) to orange (high abundance). The upper dendrogram represents the clustering of genera based on the similarity of their abundance profiles. The top color strip indicates the phylum assignment for each genus (Ascomycota, Basidiomycota, Chytridiomycota and Mucoromycota). AR, acid rain; PC, pyrochar; HC, hydrochar; PC-AR, PC combined with AR. HC-AR, HC combined with AR. CK, Control without PC, HC or AR.

To analyze the abundance patterns and clustering characteristics of soil fungal communities under severe NAR stress following BC application, a heatmap of fungal ASVs was constructed (**Figure 4**). Among the top 50 genera in relative abundance, 37 genera belonged to Ascomycota, 10 to Basidiomycota, 2 to Mucoromycota, and 1 to Chytridiomycota. In CK, fungal genera were mainly affiliated with Ascomycota and *Basidiomycota*, such as *Geosmithia*, *Parasarcopodium*, *Nomuraea*, *Hydropus*, and *Oidiodendron*, whose relative abundances were significantly higher than those in the other treatments (**Supplementary Table 3**). In AR, core fungal genera included *Gyrothrix*, *Truncatella*, *Russula*, *Thielavia*, *Septoria*, and *Hirsutella*, mostly belonging to Ascomycota and Basidiomycota, and representing characteristic functional groups of the AR fungal community. In PC-AR, key fungal genera such as *Chloridium*, *Kabatiella*, and *Bionectria* belonged to Ascomycota, whereas *Amanita* and *Clitopilus* belonged to Basidiomycota. In HC-AR, core fungal genera such as *Entoloma*, *Tomentella*, *Trechispora*, *Ganoderma*, and *Geminibasidium* belonged to Basidiomycota, while *Trichoderma*, *Stachybotrys*, and *Myrothecium* belonged to Ascomycota.

### 3.4 Effects of BC application on soil fungal α-diversity under severe NAR stress

**Figure 5:**
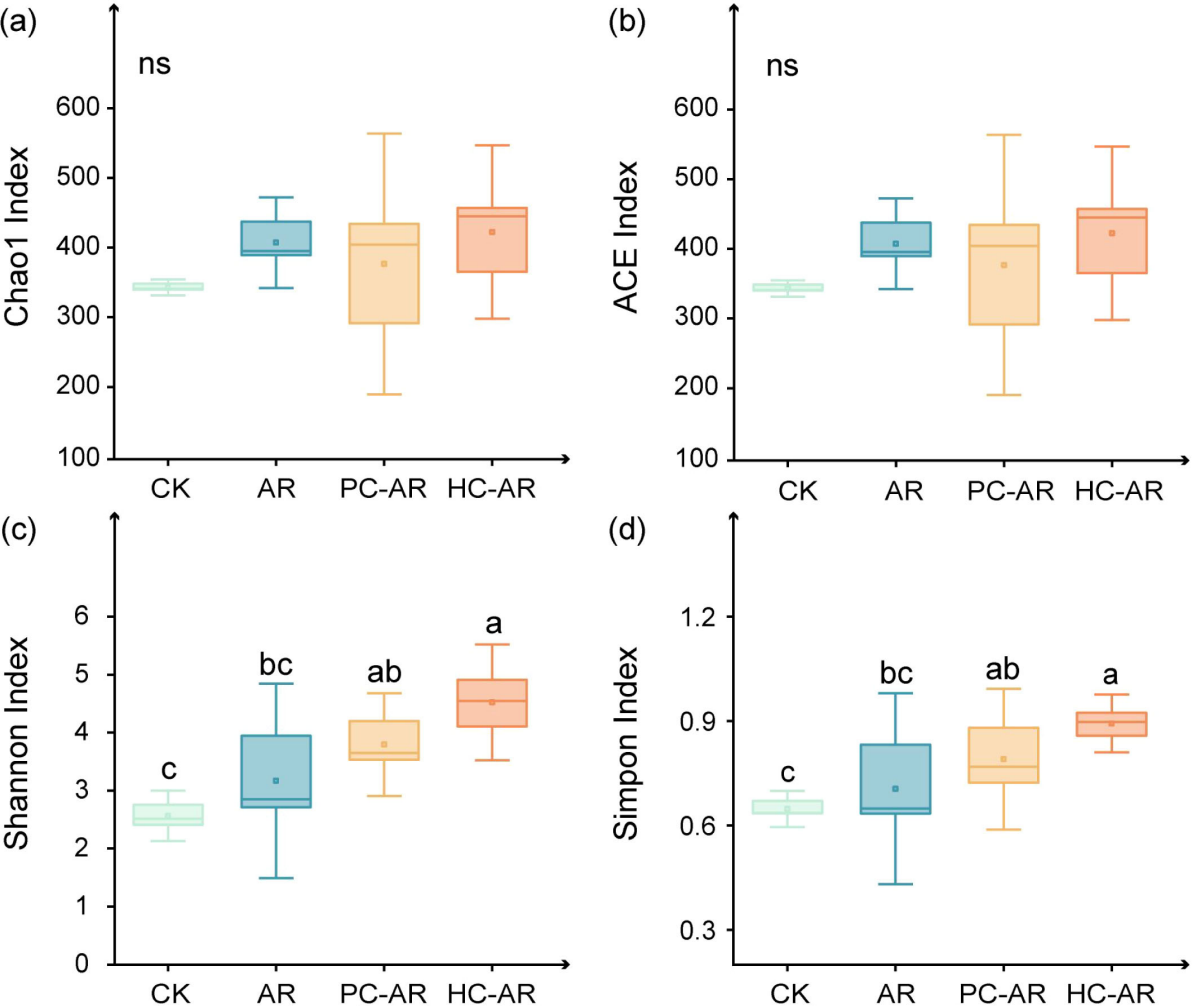
The α-diversity indices of soil fungal communities under different treatments. **(A)** Chao1 index, **(B)** ACE index, **(C)** Shannon index, and **(D)** Simpson index. Different lowercase letters above the boxes indicate significant differences among treatments based on one-way ANOVA followed by Tukey's HSD test at *P* < 0.05. "ns" indicates no significant difference (*P* > 0.05). AR, acid rain; PC, pyrochar; HC, hydrochar; PC-AR, PC combined with AR. HC-AR, HC combined with AR. CK, Control without PC, HC or AR.

**Figure 5** shows that there were no significant differences in Chao1 and ACE indices among the treatments (*P* > 0.05). For the Shannon index, HC-AR had the highest value (4.53), and the Shannon indices in PC-AR and HC-AR were significantly increased by 48.25% and 76.27%, respectively, compared with CK (*P* < 0.05). Moreover, the Shannon index in HC-AR was significantly higher than that in AR by 42.45% (*P* < 0.05). Compared with CK, the Simpson indices in PC-AR and HC-AR were significantly increased by 21.54% and 36.92%, respectively, and the Simpson index in HC-AR was also significantly higher than that in AR by 27.14% (*P* < 0.05).

**Figure 6:**
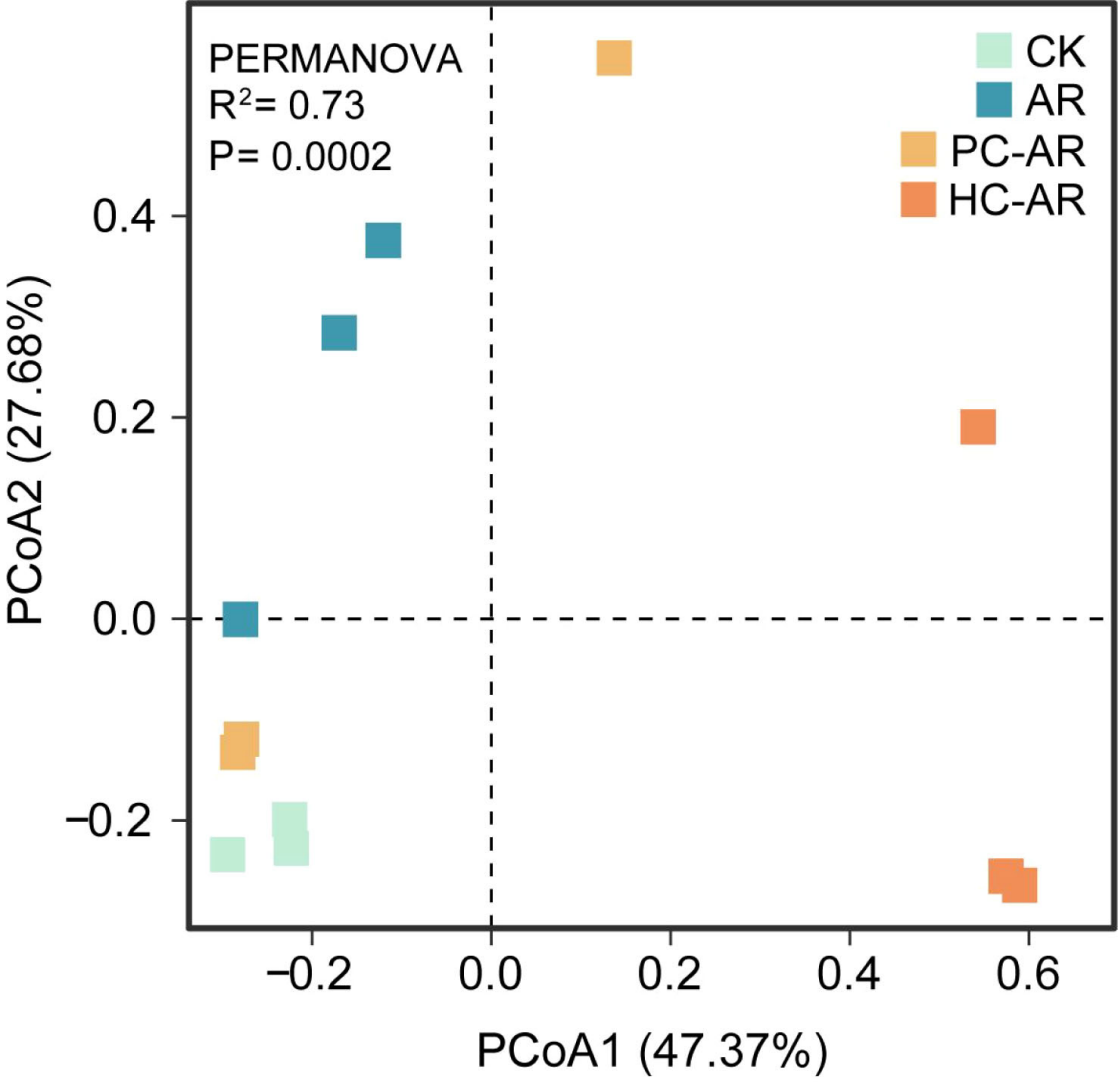
Principal coordinate analysis (PCoA) of soil fungal communities based on Bray-Curtis distances. The statistical significance of community structural differences was determined by Permutational Multivariate Analysis of Variance (PERMANOVA) based on 999 permutations. AR, acid rain; PC, pyrochar; HC, hydrochar; PC-AR, PC combined with AR. HC-AR, HC combined with AR. CK, Control without PC, HC or AR.

PCoA was used to further explore differences in soil fungal community β-diversity among treatments (**Figure 6**). The first two principal coordinates, PCoA1 and PCoA2, together explained 75.15% of the variation in fungal community structure, with PCoA1 and PCoA2 explaining 47.37% and 27.68% of the variation, respectively. PERMANOVA showed that the among-group variation had an R² of 0.73 with a significance level of *P* = 0.0002. CK samples were clustered in the negative region of PCoA1 and clearly separated from the other treatments. AR samples were mainly distributed in the negative region of PCoA1 and the middle-to-positive region of PCoA2. PC-AR samples were located in the positive region of PCoA1 and both the positive and negative regions of PCoA2, and were separated from CK and AR. HC-AR samples clustered in the high positive region of PCoA1 and the middle-to-negative region of PCoA2, with no overlap with the other treatments. The non-overlapping distribution of sample points among treatments indicated that the fungal community compositions differed significantly among treatments (*P* = 0.0002).

### 3.5 Relationships between soil fungal community structure and environmental factors

**Figure 7:**
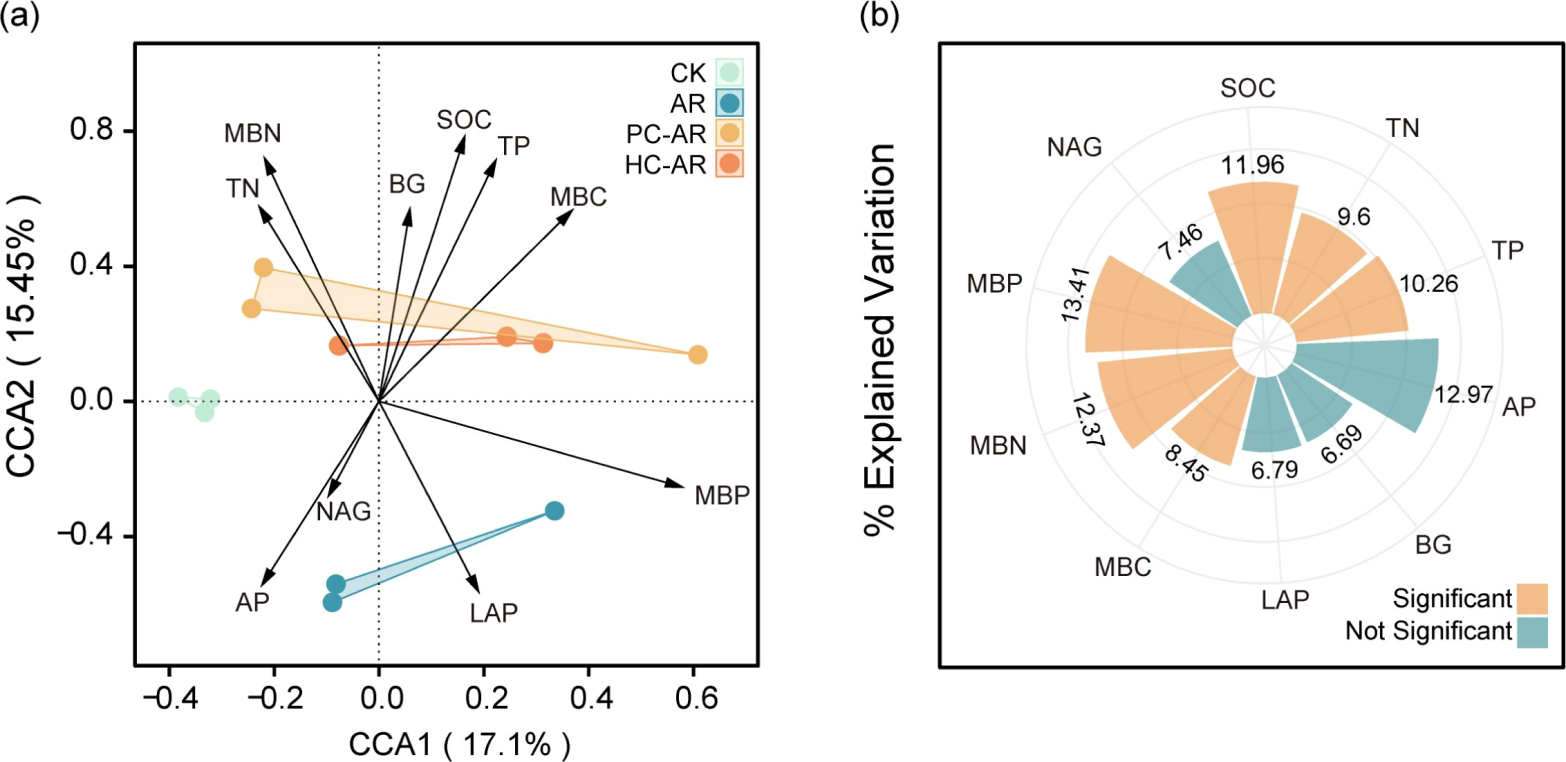
Relationships between environmental factors and fungal community structure. **(A)** The correlations between fungal community composition and soil properties. Arrows indicate the direction and magnitude of environmental vectors. **(B)** The percentage of variation in fungal community structure explained by each environmental factor. Orange bars indicate statistically significant factors (*P* < 0.05), while green bars indicate non-significant factors. SOC, soil organic carbon; TN, total nitrogen; TP, total phosphorus; MBC, microbial biomass carbon; MBN, microbial biomass nitrogen; MBP, microbial biomass phosphorus; BG, β-glucosidase; NAG, N-acetyl-β-D-glucosaminidase; LAP, leucine aminopeptidase; AP, acid phosphatase; AR, acid rain; PC, pyrochar; HC, hydrochar; PC-AR, PC combined with AR. HC-AR, HC combined with AR. CK, Control without PC, HC or AR.

Canonical correspondence analysis (CCA) of soil physicochemical properties and the relative abundances of microbial ASVs was conducted to examine the effects of different treatments on fungal community composition and the key environmental drivers (**Figure 7**). In the CCA (**Figure 7A**), CCA1 and CCA2 explained 17.1% and 15.45% of the variation, respectively, together accounting for 32.55% of the community–environment relationship. The fungal communities of the PC-AR and HC-AR treatments were clearly separated from those of CK and AR, reflecting treatment-specific community structures. SOC, TP, MBC, and BG were significantly positively correlated with the fungal communities in the PC-AR and HC-AR treatments. MBN and TN were associated with the PC-AR samples and were key drivers of their community structure, whereas soil LAP and soil AP drove the differentiation of the AR community. Among the measured soil properties, soil C, N, and P variables (MBP, MBN, SOC, TP, TN, and MBC) had statistically significant effects on fungal community structure. Their contributions to the variation in fungal community structure were 13.41%, 12.37%, 11.96%, 10.26%, 9.60%, and 8.45%, respectively (**Figure 7B**).

**Figure 8:**
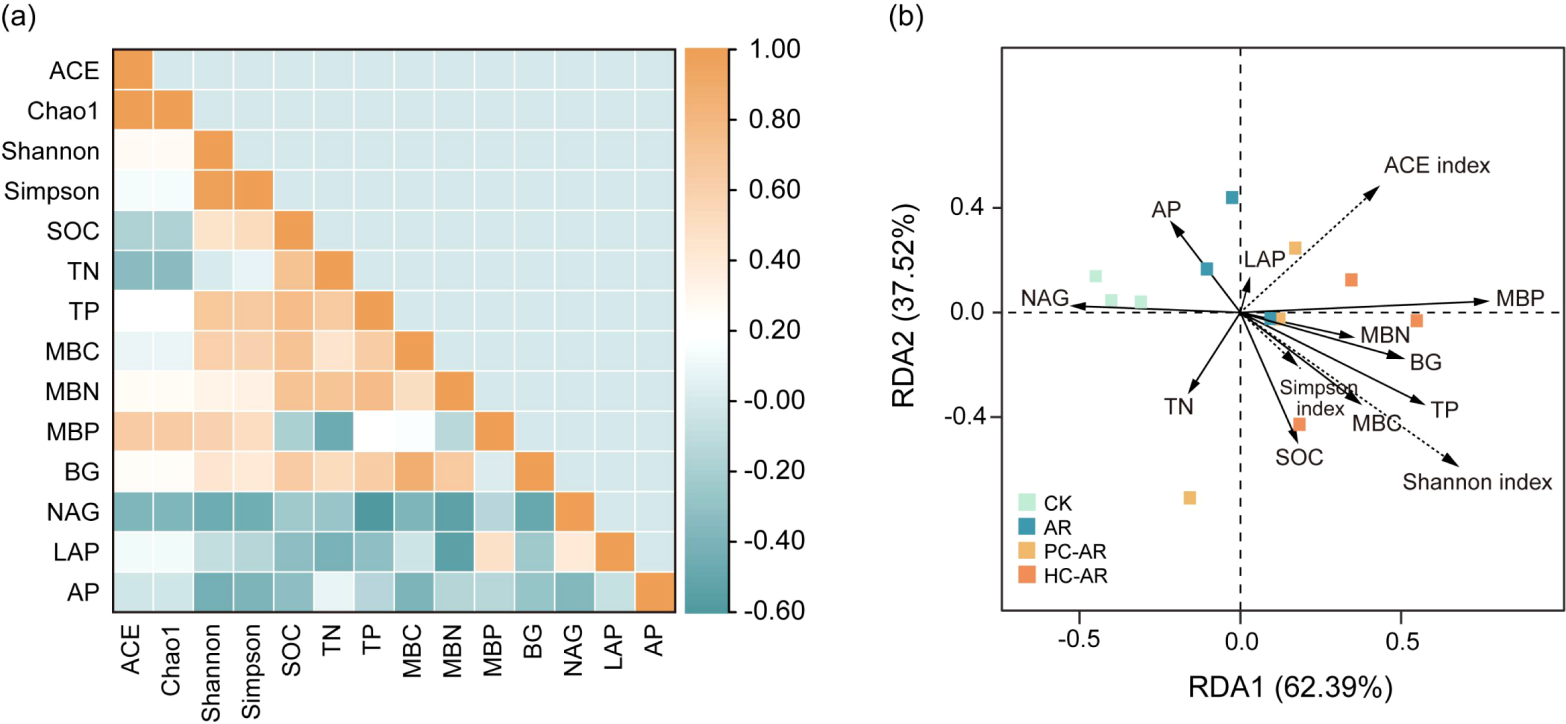
Linkages between soil fungal diversity and environmental factors. **(A)** The relationships between fungal α-diversity indices and soil properties. **(B)** The effects of environmental variables on fungal diversity. SOC, soil organic carbon; TN, total nitrogen; TP, total phosphorus; MBC, microbial biomass carbon; MBN, microbial biomass nitrogen; MBP, microbial biomass phosphorus; BG, β-glucosidase; NAG, N-acetyl-β-D-glucosaminidase; LAP, leucine aminopeptidase; AP, acid phosphatase; AR, acid rain; PC, pyrochar; HC, hydrochar; PC-AR, PC combined with AR. HC-AR, HC combined with AR. CK, Control without PC, HC or AR.

Correlation analysis between soil fungal community diversity and soil physicochemical properties under different treatments (**Figure 8A**) showed that TP, MBC, and MBP were all positively correlated with fungal richness indices (Chao1 and ACE) and diversity indices (Simpson and Shannon). MBP was significantly positively correlated with ACE, Chao1, and Shannon indices (*P* < 0.05; r = 0.65, r = 0.65, and r = 0.59, respectively). TP was significantly positively correlated with Shannon and Simpson indices (*P* < 0.05; r = 0.68 and r = 0.67, respectively). MBC was significantly positively correlated with Shannon and Simpson indices (*P* < 0.05; r = 0.61 and r = 0.59, respectively). In the RDA, the first axis (RDA1) and the second axis (RDA2) explained 62.39% and 37.52% of the variation, respectively (**Figure 8B**). In AR, the arrows for soil AP and LAP were in the same direction and showed negative correlations with α-diversity indices. In PC-AR and HC-AR, the arrow directions were highly consistent with those of ACE, Shannon, and Simpson indices. Soil fungal community diversity was significantly positively correlated with MBP (*P* < 0.05).

## 4 Discussion

### 4.1 Response of soil fungal communities to BC application under severe NAR stress

Our study found that BC application optimized the composition of soil fungal communities under severe NAR stress (**Figure 2**). At the phylum level, the relative abundance of Ascomycota increased in AR, whereas the relative abundance of Basidiomycota decreased, which is consistent with the findings of Zhou et al. (2023). Under BC treatments, the relative abundance of ectomycorrhizal fungi such as *Russula* decreased significantly, which may be attributed to the increase in SOC and available P caused by BC application (**Figure 1**). Previous studies have suggested that excessive inputs of C sources and nutrients may inhibit the growth of symbiotic fungi such as ectomycorrhizal fungi (Peng et al., 2024). Notably, the relative abundance of acid-sensitive ectomycorrhizal fungi such as *Tomentella* increased markedly in the BC treatments. This may be because soil acidification created an unfavorable environment for *Tomentella* under NAR stress, whereas BC alleviated soil acidification and likely provided a more suitable environment for its colonization and growth, thereby increasing its relative abundance (Baldrian et al., 2022; Li et al., 2023b).

Moreover, BC application significantly enhanced soil fungal community diversity under severe NAR stress. Compared with AR, both types of BC treatments increased the Shannon index (**Figure 5**), which is consistent with the findings of Li et al. (2020) that BC may buffer soil acidity and increase available C sources, thereby providing more ecological niches for different fungi and promoting soil fungal α-diversity. Increases in the Simpson index also reflected an improvement in community evenness, which may be because BC reduced the competitive advantage of acid-tolerant dominant groups represented by *Russulales*, allowing previously disadvantaged genera such as *Tomentella* and *Penicillium* to expand (Chen et al., 2019). Our study found that soil fungal communities in AR differed markedly from those in CK, and BC application further reshaped the soil fungal community structure under NAR stress. Functional guild analysis revealed that severe NAR led to functional homogenization dominated by ectomycorrhizal fungi, whereas BC application restored saprotrophic and mixed-nutrient guilds (**Supplementary Figure 2**). This indicates that NAR stress caused significant changes in soil fungal community structure and function, and that BC may help mitigate the disturbance of soil fungal communities induced by NAR stress (Wang et al., 2020).

### 4.2 Differential effects of PC and HC on soil fungal communities

Under severe NAR stress, HC and PC differentially restructured the soil fungal communities in the plantation. The total number of fungal ASVs and the number of unique ASVs in HC-AR were higher than those in the other treatments (**Figure 2**), which is consistent with previous findings that HC has a significant advantage in broadening fungal niche width and enhancing the environmental adaptability of communities (Kravchenko et al., 2024; Watson et al., 2021). At the phylum level, PC-AR increased the relative abundance of Basidiomycota, whereas HC-AR significantly reduced it (**Figure 2**). This difference might be attributed to the material properties of PC and HC. Compared with HC, PC retains a larger proportion of lignin-dominated, stable C skeletons, which is more conducive to the growth and colonization of Basidiomycota that use lignin as their main substrate (Zhou et al., 2023). In contrast, HC, as an input of highly labile C, strongly stimulated the activity of r-strategist fungi (e.g., *Trichoderma* and *Penicillium*), which likely weakened the competitive advantage of Basidiomycota that require specific substrates (**Figure 3**) (Kambo and Dutta, 2015). The higher MBC content and BG activity in HC-AR than those in PC-AR also support the view that HC input significantly promoted microbial metabolic activity and C cycling processes (**Figure 1**).

Compared with PC-AR, HC-AR had a more pronounced effect on increasing soil fungal α- and β-diversity (**Figures 5**, **6**). This might be closely associated with the unique physicochemical properties and higher bioavailability of HC (Watson et al., 2021). First, HC input not only effectively compensated for SOC losses caused by AR stress, but also provided easily utilizable C sources and attachment microenvironments for fungi owing to its surface rich in oxygen-containing functional groups (Watson et al., 2021), thereby facilitating the colonization and expansion of diverse fungal taxa. Second, HC enhanced the availability of N and P in soil, promoting the rapid proliferation and niche occupation of non-dominant species, mainly Ascomycota (Muneer et al., 2021). By contrast, PC, with its highly aromatic and structurally stable C framework, has relatively low biodegradability and can be utilized only by a limited number of Basidiomycota with the capacity to degrade recalcitrant C. As a result, the activation range and rate of PC on fungal communities are narrower and slower (Bolan et al., 2024; Kravchenko et al., 2024). On this basis, we speculate that PC is more suitable for long-term ecological restoration, whereas HC has greater potential for short-term nutrient activation (Luo et al., 2023; Sun et al., 2022). Nevertheless, this inference requires further verification through long-term experiments.

### 4.3 Relationships between soil fungal community structure and environmental factors induced by BC application under severe NAR stress

CCA analysis showed that the variation in soil fungal community composition induced by BC application was mainly jointly driven by MBN and MBP, as well as C and N nutrient status under severe NAR stress. Nevertheless, soil fungal community composition was more strongly driven by extracellular enzymes (e.g., LAP and AP) under NAR stress (**Figure 7**). This may be attributed to the ability of BC to regulate soil nutrient pools and microbial biomass, thereby driving microbial community succession (Dai et al., 2021). The RDA analysis further indicated that fungal diversity was positively influenced by MBP and TP (**Figure 8**). Notably, although MBP increased significantly in the AR treatment (**Figure 1**), fungal diversity did not show a corresponding increase (**Figure 5**). This may be explained by the fact that short-term severe NAR induced the lysis of some fungal cells (Tian et al., 2021), and the released intracellular P was rapidly immobilized by the surviving microorganisms (Hu et al., 2022). It might result in a transient MBP increase without a concurrent improvement in fungal diversity over the short term. Therefore, future studies should continuously monitor the dynamics of fungal diversity to clarify the long-term effects.

**Figure 9:**
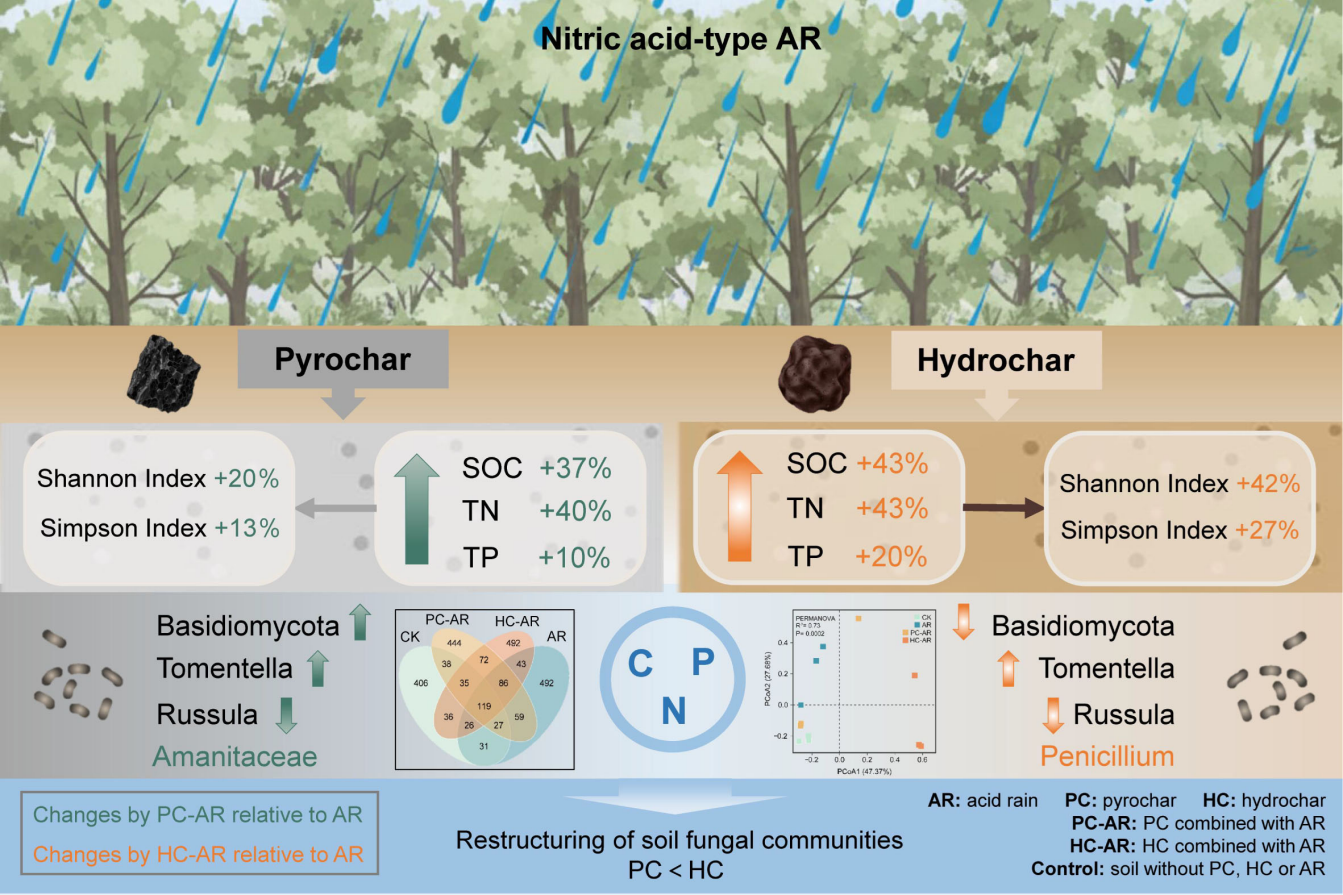
The conceptual diagram of hydrochar and pyrochar effects on soil fungal community in *Quercus acutissima* plantations under severe nitric acid-type acid rain stress.

Meanwhile, severe NAR significantly intensified the loss of soil organic matter and nitrogen (Zhou et al., 2023), whereas BC input significantly increased key nutrients such as SOC and TN (**Figure 9**). This might provide a resource-rich microenvironment for the survival and reproduction of multiple fungal groups, and promote species complementarity and coexistence (Bolan et al., 2024). Soil BG activity was significantly enhanced under HC-AR, indicating that the fungal community was in a state of high C metabolism and could efficiently decompose and utilize substrate C, thereby markedly increasing MBC and soil fungal diversity in the short term (Kambo and Dutta, 2015). With the simultaneous improvement of C, N, and P, environmental filtering pressure was substantially weakened, allowing nutrient-sensitive taxa with strong resource acquisition capacity to expand and participate in community reassembly (Xia et al., 2023). This suggests that HC exhibited more pronounced ecological effects than PC in alleviating severe NAR stress and promoting the recovery and reconstruction of soil fungal communities.

### 4.4 Limitations and future perspectives

Under severe NAR stress, both PC and HC significantly improved the structure of soil fungal communities in *Q. acutissima* plantations by modulating essential chemical properties of acidified soil. These findings demonstrate that BC amendment can effectively mitigate the negative impact of intense NAR on soil fungal communities, providing a scientific basis for the ecological restoration of plantation ecosystems in NAR-affected regions and for the resource-oriented utilization of BC.

Several limitations of this study should be acknowledged. (1) The background soil pH in the experimental area was relatively low. Further studies conducted in soils with different initial properties are needed to verify the broader applicability of our conclusions. (2) A control group amended with BC only could be added, which would help distinguish the direct effects of BC on fungal communities from its interactive effects under NAR stress, thereby providing stronger evidence for the role of BC in alleviating NAR stress. (3) The long-term ecological effects of the interaction between BC and acid deposition require validation through sustained field monitoring at permanent observation stations. (4) Future research could integrate metagenomic approaches to simultaneously profile multiple microbial groups (e.g., fungi, bacteria, and actinomycetes), thereby offering a more systematic understanding of the mechanisms through which BC regulates soil micro-ecology.

## 5 Conclusion

Under severe nitric acid-type acid rain (NAR) stress, biochar (BC) application significantly altered the fungal community structure in *Quercus acutissima* plantation soils by improving soil physicochemical properties, with distinct effects observed between pyrochar (PC) and hydrochar (HC). In PC-amended soils, the relative abundance of Basidiomycota increased, whereas HC amendment promoted the relative abundances of Ascomycota (e.g., *Penicillium*) and specific ectomycorrhizal fungi (e.g., *Tomentella*). Both PC and HC enhanced fungal community diversity, with the recovery of fungal α-diversity primarily driven by the combined increases in microbial biomass and soil organic carbon. Compared with PC, HC exhibited superior performance and greater short-term potential in restoring the soil micro-environment. These findings provide a scientific basis for the ecological restoration of environments affected by severe acid deposition and support the sustainable development of circular agroforestry systems through the targeted use of BC.

## Data Availability

The original contributions presented in the study are publicly available. This data can be found here: https://ngdc.cncb.ac.cn/bioproject/browse/PRJCA059488.

## Supplementary material

Supplementary Figure 1 Shows the Scanning Electron Microscope micrographs of pyrochar and hydrochar.

Supplementary Figure 2 Shows the functional guild analysis of soil fungal communities under different treatments.

Supplementary Table 1 Shows the basic properties of pyrochar and hydrochar.

Supplementary Table 2 Shows statistics of unique and shared ASVs among different treatments.

Supplementary Table 3 Shows the relative abundance of dominant fungal phyla in different treatments.
