# Supplementary material for: Hydrochar and pyrochar enhanced soil fungal community diversity in a Quercus acutissima plantation under severe nitric acid-type acid rain stress
Source: Front Plant Sci. 2026 May 28;17:1821765. doi: 10.3389/fpls.2026.1821765 (PMC13255048; doi:10.3389/fpls.2026.1821765)
Supplement: Supplementary Figure 1 — Shows the Scanning Electron Microscope micrographs of pyrochar and hydrochar. [file SupplementaryFile1.docx]

**Supplementary material for**

Hydrochar and pyrochar enhanced soil fungal community diversity in a *Quercus acutissima* plantation under severe nitric acid-type acid rain stress

Yan Wang^a, #^, Shushu Yao^a, #^, Haibo Hu^a, *^, Xiaopeng Xu^a^, Xiaoxiao Liu^d^, Peng Cui^a, c, *^, Danyan Chen^b^, Yuanhao Liu^a^, Yuanyuan Feng^a,c *^

**a** Co-Innovation Center for Sustainable Forestry in Southern China, College of Forestry and Grassland, Nanjing Forestry University, Nanjing 210037, China

**b** College of Horticulture, Jinling Institute of Technology, Nanjing 210038, China

**c** Department of Applied Physics and Electronics, Umea University, Umea 90187, Sweden

**d** School of Agriculture, Yunnan University, Kunming, 650504, China

^#^ These authors contributed equally to this paper.

^*^Corresponding author. Tel.: +86-25-84391526;

E-mail: [huhaibo@njfu.edu.cn;](mailto:huhaibo@njfu.edu.cn) cui.peng@umu.se; Feng.Yuanyuan@hotmail.com

**
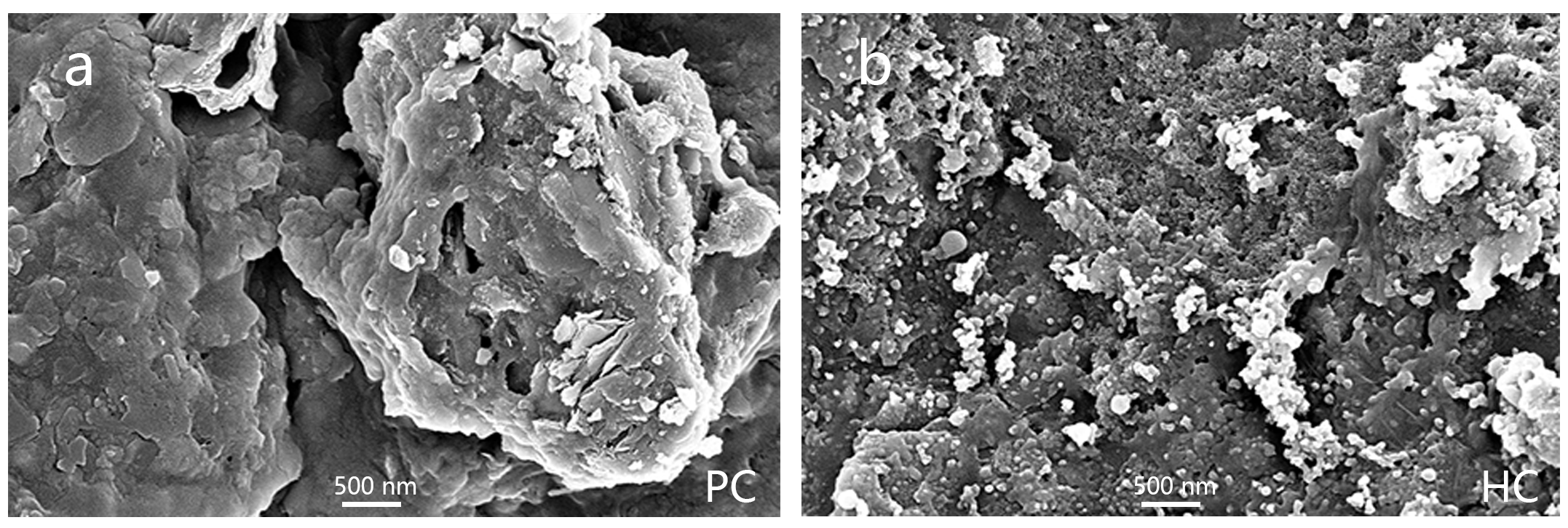
**

**Figure S1** The Scanning electron microscope micrographs of pyrochar (PC, a) and hydrochar (HC, b).


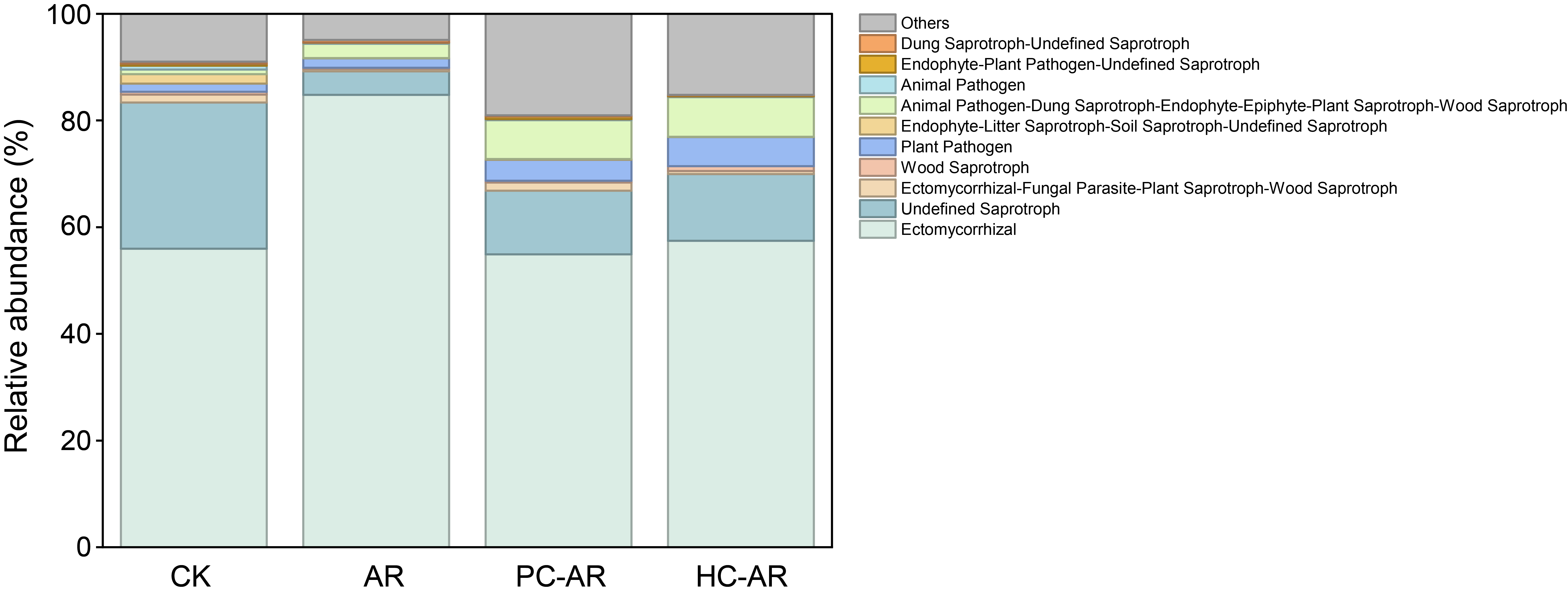


**Figure S2** The functional guild analysis of soil fungal communities under different treatments. AR: acid rain; PC: pyrochar; HC: hydrochar; PC-AR: PC combined with AR. HC-AR: HC combined with AR. CK: control without PC, HC or AR.

**Table S1** The physicochemical properties of pyrochar (PC) and hydrochar (HC). BC: Biochar; OC: Organic carbon; TN: Total nitrogen; SSA: Specific surface area; PD: Pore diameter; PV: Pore volume.

| BC type | pH | Ash  (%) | O/C | H/C | OC  (g kg^-1^) | TN  (g kg^-1^) | TP  (g kg^-1^) | K  (g kg^-1^) | Ca  (g kg^-1^) | Fe  (mg kg^-1^) | Mn  (mg kg^-1^) | SSA  (m^2^ g^-1^) | PD  (nm) | PV  (cm^3^ g^-1^) |
| --- | --- | --- | --- | --- | --- | --- | --- | --- | --- | --- | --- | --- | --- | --- |
| PC | 10.0 | 11.2 | 0.08 | 0.42 | 589.8 | 4.6 | 0.8 | 5.7 | 8.4 | 230.1 | 193.0 | 1.80 | 1.36 | 0.008 |
| HC | 5.6 | 16.5 | 0.32 | 0.88 | 450.6 | 5.3 | 1.1 | 6.8 | 6.9 | 312.3 | 219.2 | 3.26 | 1.34 | 0.010 |

**Table S2** Statistics of unique and shared ASVs among different treatments. AR: acid rain; PC: pyrochar; HC: hydrochar; PC-AR: PC combined with AR. HC-AR: HC combined with AR. CK: control without PC, HC or AR. ASV, amplicon sequence variant.

| Pattern | ASV_number |
| --- | --- |
| Total detected ASVs | 1175 |
| CK | 406 |
| AR | 492 |
| PC-AR | 444 |
| HC-AR | 492 |
| CK & AR & PC-AR & HC-AR | 119 |
| AR & PC-AR & HC-AR | 86 |
| PC-AR & HC-AR | 72 |
| AR & PC-AR | 59 |
| AR & HC-AR | 43 |
| CK & PC-AR | 38 |
| CK & HC-AR | 36 |
| CK & PC-AR & HC-AR | 35 |
| CK & AR | 31 |
| CK & AR & PC-AR | 27 |
| CK & AR & HC-AR | 26 |

**Table S3** Relative abundance of dominant fungal phyla in different treatments. Values indicate the relative abundance (%) of each phylum in the corresponding treatment. AR: acid rain; PC: pyrochar; HC: hydrochar; PC-AR: PC combined with AR. HC-AR: HC combined with AR. CK: control without PC, HC or AR.

| Phylum | CK | AR | PC-AR | HC-AR |
| --- | --- | --- | --- | --- |
| Basidiomycota | 29.51% | 26.80% | 32.40% | 11.30% |
| Ascomycota | 24.76% | 29.70% | 6.24% | 39.30% |
| Mucoromycota | 28.41% | 26.82% | 15.85% | 28.91% |
| Chytridiomycota | 0.00% | 0.00% | 18.75% | 81.25% |
| Rozellomycota | 0.00% | 0.00% | 0.00% | 100.00% |
| Glomeromycota | 0.00% | 0.00% | 100.00% | 0.00% |
| Other | 11.27% | 16.15% | 15.48% | 57.10% |
